# Dual titration of minute ventilation and sweep gas flow to control carbon dioxide variations in patients on venovenous extracorporeal membrane oxygenation

**DOI:** 10.1186/s13613-023-01138-5

**Published:** 2023-05-25

**Authors:** Paul Masi, François Bagate, Samuel Tuffet, Mariantonietta Piscitelli, Thierry Folliguet, Keyvan Razazi, Nicolas De Prost, Guillaume Carteaux, Armand Mekontso Dessap

**Affiliations:** 1 Service de Médecine Intensive Réanimation, AP-HP, Hôpitaux Universitaires Henri-Mondor, 94010 Créteil, France; 2 CARMAS, Univ Paris Est Créteil, 94010 Créteil, France; 3 IMRB, Univ Paris Est Créteil, INSERM, 94010 Créteil, France; 4 Service de chirurgie cardiaque, DMU CARE, Assistance Publique-Hôpitaux de Paris (AP-HP), Hôpitaux Universitaires Henri Mondor, Université Paris Est Créteil, Faculté de Santé, F-94010 Créteil, France

**Keywords:** Extracorporeal membrane oxygenation, Carbon dioxide control, Titration sweep gas flow, Titration minute ventilation, Ultra-protective ventilation, Intracranial bleeding

## Abstract

**Background:**

The implantation of venovenous extracorporeal membrane oxygenation (VV-ECMO) support to manage severe acute respiratory distress syndrome generates large variations in carbon dioxide partial pressure (PaCO_2_) that are associated with intracranial bleeding. We assessed the feasibility and efficacy of a pragmatic protocol for progressive dual titration of sweep gas flow and minute ventilation after VV-ECMO implantation in order to limit significant PaCO_2_ variations.

**Patients and methods:**

A protocol for dual titration of sweep gas flow and minute ventilation following VV-ECMO implantation was implemented in our unit in September 2020. In this single-centre retrospective before-after study, we included patients who required VV-ECMO from March, 2020 to May, 2021, which corresponds to two time periods: from March to August, 2020 (control group) and from September, 2020 to May, 2021 (protocol group). The primary endpoint was the mean absolute change in PaCO_2_ in consecutive arterial blood gases samples drawn over the first 12 h following VV-ECMO implantation. Secondary endpoints included large (> 25 mmHg) initial variations in PaCO_2_, intracranial bleedings and mortality in both groups.

**Results:**

Fifty-one patients required VV-ECMO in our unit during the study period, including 24 in the control group and 27 in the protocol group. The protocol was proved feasible. The 12-h mean absolute change in PaCO_2_ was significantly lower in patients of the protocol group as compared with their counterparts (7 mmHg [6–12] vs. 12 mmHg [6–24], *p* = 0.007). Patients of the protocol group experienced less large initial variations in PaCO_2_ immediately after ECMO implantation (7% vs. 29%, *p* = 0.04) and less intracranial bleeding (4% vs. 25%, *p* = 0.04). Mortality was similar in both groups (35% vs. 46%, *p* = 0.42).

**Conclusion:**

Implementation of our protocol for dual titration of minute ventilation and sweep gas flow was feasible and associated with less initial PaCO_2_ variation than usual care. It was also associated with less intracranial bleeding.

**Supplementary Information:**

The online version contains supplementary material available at 10.1186/s13613-023-01138-5.

## Background

In patients with refractory acute respiratory distress syndrome (ARDS), venovenous extracorporeal membrane oxygenation (VV-ECMO) [1] provides lung-protective ventilation, improves gas exchange, and maintains arterial partial pressure of carbon dioxide (PaCO_2_) and oxygen (PaO_2_) stable by adjusting the rates of sweep gas flow (SGF) to the oxygenator and VV-ECMO blood flow, respectively [2].

After VV-ECMO initiation, recommendations and expert consensus suggest switching to ultra-protective ventilation, defined by a plateau pressure ≤ 24 cmH_2_O, a positive end-expiratory pressure (PEEP) ≥ 10 cmH_2_O, and a respiratory rate between 10 and 20 breaths per minute[3–5]. This switch can lead to a significant drop in PaCO_2_, especially if the SGF is not well balanced by an adequate decrease in ventilator minute ventilation (V_M_) [3, 5]. In addition, this switch could be challenging if ECMO was implanted by a mobile circulatory assistance team followed by ambulance transport to a tertiary hospital.

The cerebral vascular tone is highly sensitive to changes in PaCO_2_ [6]. For instance, a fast drop in PaCO_2_ within the first hours following VV-ECMO initiation is associated with an increased risk of cerebral haemorrhage [7–9], a dreaded technique-related complication [10]. Upon VV-ECMO initiation, it is recommended to set the SGF rate at 2 L/min, and titrate frequently to ensure slow, controlled modulation of PaCO_2_ and pH [5]. To date, no specific procedures for dual adjustment of oxygenator SGF and V_M_ have been reported. Besides, this “frequent titration” is potentially difficult to perform in clinical settings, especially when VV-ECMO is implanted by mobile circulatory assistance teams.

Based on the physiological reasoning that a decrease in V_M_ and an increase in oxygenator SGF have opposite effects on arterial PaCO_2_, we implemented a pragmatic protocol for progressive dual titration of SGF and V_M_ in our unit. The protocol objective was to limit PaCO_2_ variations the time needed to achieve ultra-protective ventilation in the first 12 h of VV-ECMO. The present study was conducted to assess the feasibility and efficacy of such a protocol.

## Patients and methods

### Patients

Consecutive patients requiring VV-ECMO in the medical ICU of a tertiary university hospital hosting a mobile circulatory assistance unit were retrospectively included between March, 2020 and May, 2021 in this before-after study. Trained cardiovascular surgeons performed all procedures at the bedside, in our referral ICU or in other ICUs of our network (projection as a mobile circulatory assistance team). The criteria for VV-ECMO implantation were those used in EOLIA trial [4]. Our protocol for dual titration of SGF and V_M_ was implemented in September 2020, allowing us to define two groups of patients according to the two periods: “control group” (before) from March to August, 2020 (i.e. settings of SGF and V_M_ were at the clinicians’ discretion, with no standardised procedure), and “protocol group” (after) from September, 2020 to May, 2021 (i.e. physicians were incited to follow a formal dual titration protocol for patients admitted during that period). The follow up ended at hospital discharge or death. Protocol adherence was considered good if the blood gas was sampled at the expected time and if the expected decrease in V_M_ (− 1500 mL/min) was performed with a tolerated error of 20% (− 1200 to − 1800 mL/min). In both groups (control and protocol), a bolus of 4000 IU of unfractionated heparin was infused at the time of ECMO implantation. During the ECMO course, patients were still given unfractionated heparin intravenously to maintain activated partial thromboplastin time (aPTT) at 1–1.5 times the normal, and anti-Xa activity between 0.2–0.3 IU/ml [3]. Heparin dose was adjusted at least once a day according to aPTT level and anti-Xa activity. Owing to the changes in care standards and recommendations [3, 11] during COVID-19 outbreak, the targeted level of anti-Xa activity was set at 0.3–0.5 IU/ml for part of the study.

### Dual titration protocol

**Fig. 1:**
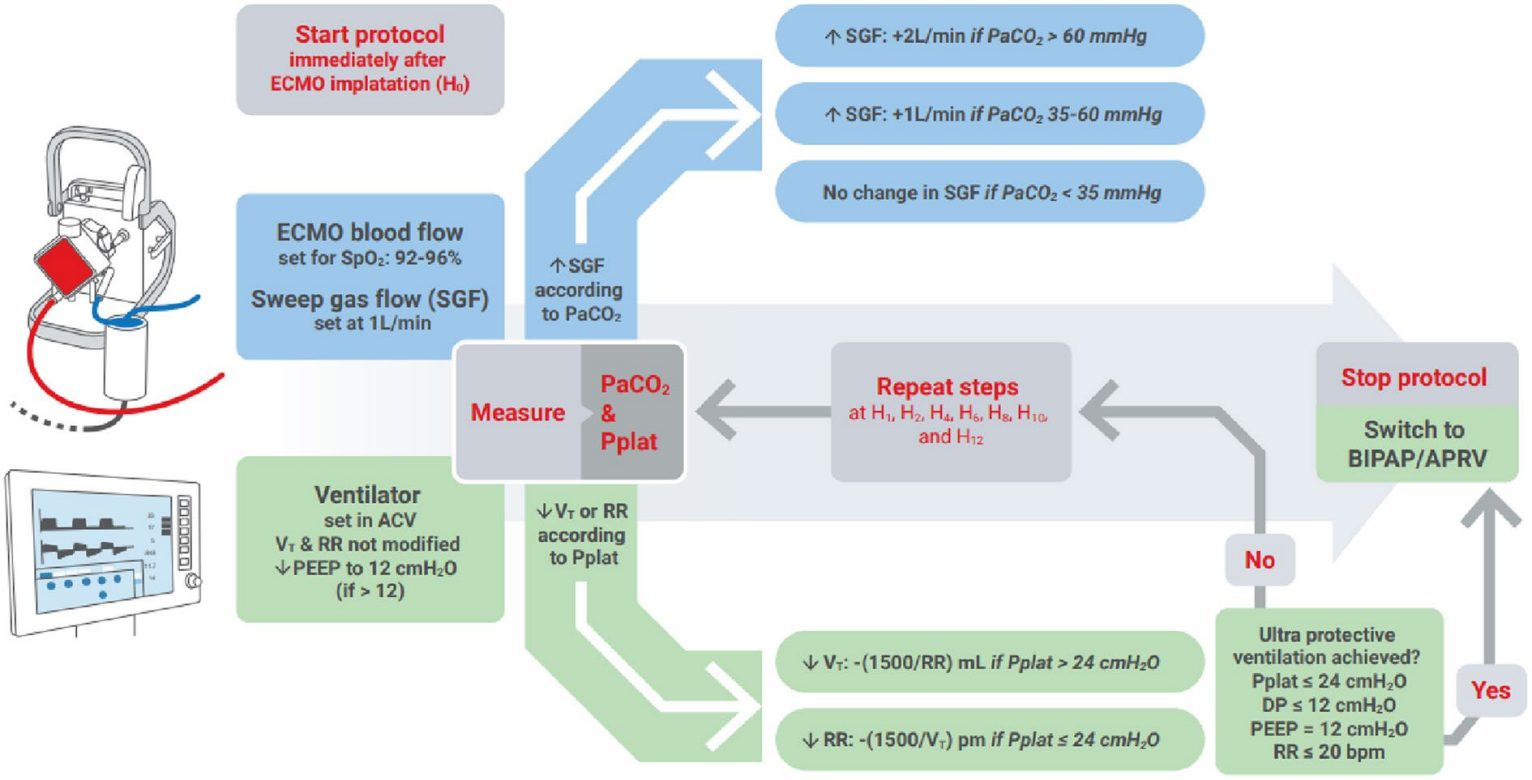
Dual titration protocol. *ECMO* extracorporeal membrane oxygenation; *SpO2* oxygen saturation in peripheral blood; *ACV* assist control ventilation mode; *PaCO2* partial pressure of carbon dioxide in arterial blood; *Pplat* plateau pressure; *DP* driving pressure; *BIPAP/APRV* bi-level positive airway pressure/airway pressure release ventilation; *VT* tidal volume; *RR* respiratory rate; *pm* breaths per minute; *PEEP* positive end-expiratory pressure

The entire dual titration protocol is available in Fig. 1, and the bedside copy in Additional data (Additional file 1: Table S1, also available with automatic calculation in an Excell sheet "cite the excell sheet as an additional file"). An example of the protocol is displayed in Additional file 1: Table S2. Briefly, the protocol included the following steps: immediately after ECMO implantation, initial SGF was set at 1 L/min, and ECMO blood flow rate was adjusted by the perfusionist to attain a pulse oximetry saturation between 92 and 96%; the ventilator was set in volume assist control mode (ACV), and the positive end-expiratory pressure (PEEP) was kept at 12 cmH_2_O by the intensivist if initially it was higher than 12 cmH_2_O; the other settings were kept unchanged. If ECMO was implanted by our mobile circulatory assistance team in a primary ICU, parameters were left in this configuration (SGF rate at 1 L/min, ECMO blood flow for a pulse oximetry between 92 and 96%, and PEEP lowered to 12 cmH_2_O, without other modification) until transfer to our referral ICU where its intensivist would follow the rest of the protocol. V_M_ was then reduced by steps of 1.5 L/min by first lowering the tidal volume to target a plateau pressure ≤ 24 cmH_2_O, then decreasing the respiratory rate down to 20/min or less. At each step, SGF was simultaneously increased by 1 L/min if PaCO_2_ was ≥ 35 and ≤ 60 mmHg, 2 L/min if PaCO_2_ > 60 mmHg, or left unchanged if PaCO_2_ < 35 mmHg. We would switch the ventilator mode from ACV to bi-level positive airway pressure/airway pressure release ventilation (BIPAP/APRV) as soon as the ultra-protective ventilation was reached (plateau pressure ≤ 24 cmH_2_O, PEEP ≤ 12 cmH_2_O, and respiratory rate ≤ 20/min). The initial settings of BIPAP/APRV were a lower pressure of 12 cmH_2_O and a higher pressure of 24 cmH_2_O, secondarily adapted to patient’s clinical condition. For PaCO_2_ measurement, ABG was performed at each step and at least 15 min after any adjustment of VM or SGF. The titration steps were to be performed at the following timeframes: one hour after ECMO implantation (H1), then at two, and every two hours until switching to ultra-protective ventilation with which the protocol was stopped.

### Endpoints

The primary endpoint was the mean absolute change in PaCO_2_ detected on consecutive arterial blood gases samples drawn around VV-ECMO implantation time point (including the last sampling before VV-ECMO and the consecutive samplings over the first 12 h after). For example, for a patient switched to ultra-protective ventilation at H10, the mean absolute change in PaCO_2_ was calculated as follows: (│PaCO_2_preECMO—PaCO_2_H0│ + │PaCO_2_H0—PaCO_2_H1│ + │PaCO_2_H1—PaCO_2_H2│ + │PaCO_2_H2—PaCO_2_H4│ + │PaCO_2_H4—PaCO_2_H6│ + │PaCO_2_H6—PaCO_2_H8 │ + │PaCO_2_H8—PaCO_2_H10│)/7. Secondary endpoints were the percentage of patients with large initial variations in PaCO_2_ (i.e. with │PaCO_2_preECMO—PaCO_2_H0│ > 25 mmHg), feasibility and adherence to the protocol, bleeding (intracranial and other sites, as per the international Society on Thrombosis and Haemostasis definition) [12], morbidity, and hospital mortality. We considered the protocol feasible if more than 75% of the arterial blood gases were taken at the right time and if more than 75% of titration of minute ventilation and sweep gas flow were in accordance with the protocol.

### Statistical analyses

Statistical analyses were performed using JMP software (version 14; SAS Institute Inc., Cary, NC). Categorical variables are presented as number and percentages. Fisher exact test or *χ*^2^ were used for between-group comparisons, as appropriate. Continuous variables are expressed as median and interquartile range and were compared using Mann–Whitney *U* test.

## Results

### Patients

From March, 2020 to May, 2021, 51 patients required VV-ECMO in our ICU to manage ARDS that was caused in all patients by SARS-CoV-2 pneumonia with no other viral coinfection. Twenty-four patients were included in the control group and 27 in the protocol group. Description of patients’ characteristics is provided in Table 1. Briefly, patients included in the protocol group were younger, more often female and obese, and exhibited a trend toward higher SAPS 2 score. In addition, more patients in the protocol group were implanted by a mobile circulatory assistance team. Ventilator settings and arterial blood gases before and after VV-ECMO implantation are reported in Table 2. Settings and gas exchange were similar between the two groups before VV-ECMO implantation.

**Table 1:** Clinical characteristics of patients

Variables	All patients (*n* = 51)	Control group (*n* = 24)	Protocol group (*n* = 27)	*p*
Age	53 [45–58]	56 [50–58]	50 [43–56]	0.03
Male gender (%)	34 (67)	21 (88)	13 (48)	0.003
Body mass index (kg/m^2^)	31 [27–35]	29 [26–32]	32 [30–39]	0.006
SAPS 2 at ICU admission	35 [29–53]	33 [27–46]	49 [29–57]	0.05
Comorbidities				
Diabetes mellitus	14 (27)	8 (33)	6 (22)	0.37
Arterial hypertension	25 (49)	15 (63)	10 (37)	0.07
Ischaemic heart disease	1 (2)	1 (4)	0 (0)	0.47
Chronic renal insufficiency	2 (4)	1 (4)	1 (4)	1
COPD	1 (2)	1 (4)	0 (0)	0.47
Immunodeficiency	3 (6)	2 (8)	1 (4)	0.60
Atrial fibrillation	2 (4)	2 (8)	0 (0)	0.22
Sickle cell disease	1 (2)	1 (4)	0 (0)	0.47
Smoker	8 (16)	6 (25)	2 (16)	0.13

*SAPS 2*: Simplified Acute Physiology Score 2; *ICU* intensive care unit; *COPD* chronic obstructive pulmonary disease

**Table 2:** Respiratory settings and arterial blood gases

Variables	All patients (*n* = 51)	Control group (*n* = 24)	Protocol group (*n* = 27)	*p*
Ventilation parameters before VV-ECMO implantation				
Tidal volume, mL/kg PBW	5.9 [5.3–6.6]	5.8 [5.4–6.9]	5.9 [5.1–6.3]	0.5
Positive end-expiratory pressure, cmH_2_O	10 [8–12]	10 [8–12]	10 [7–12]	0.8
Driving pressure, cmH_2_O	19 [18–24]	19 [18–21]	23 [18–26]	0.18
Respiratory rate, breaths/min	35 [30–35]	35 [30–35]	34 [25–37]	0.8
Plateau pressure, cmH_2_O	31 [29–32]	30 [28–32]	31 [29–35]	0.23
Arterial blood gases before VV-ECMO implantation				
PaO_2_/FiO_2_, mmHg	69 [59–78]	73 [59–79]	66 [57–74]	0.36
pH	7.3 [7.2–7.38]	7.3 [7.22–7.37]	7.34 [7.23–7.39]	0.38
PaCO_2_, mmHg	57 [48–67]	62 [50–80]	55 [47–63]	0.18
Lactates, mmol/L	1.4 [1–1.8]	1.4 [1.2–1.8]	1.4 [1–1.8]	0.68
Ventilation parameters after VV-ECMO implantation (H1)				
Tidal volume, mL/kg PBW	4.5 [2.6–5.9]	2.9 [1.7–4.3]	5.6 [4.5–6.2]	0.0001
PEEP, cmH_2_O	12 [10–12]	12 [12–12]	12 [8–12]	0.4
Driving pressure, cmH_2_O	14 [12–19]	12 [12–12]	17 [14–23]	0.0001
Respiratory rate, breaths/min	25 [20–35]	23 [20–34]	26 [20–35]	0.17
Plateau pressure, cmH_2_O	24 [24–30]	24 [24–24]	29 [25–34]	0.001
Arterial blood gases after VV-ECMO implantation (H1)				
pH	7.32 [7.23–7.39]	7.36 [7.22–7.42]	7.31 [7.23–7.37]	0.47
Lactates, mmol/L	1.5 [1.1–2.3]	1.5 [1–1.6]	1.6 [1.1–2.7]	0.26
PaCO_2_, mmHg	49 [42–59]	53 [42–58]	49 [41–59]	0.84
PaCO_2_ variations				
Maximal absolute change just after ECMO > 25 mmHg^a^, n (%)	9 (18)	7 (29)	2 (7)	0.04
Mean absolute change within the 12 h following ECMO implantation^b^, mmHg	8 [6–13]	12 [6–24]	7 [6–10]	0.007
Ventilation parameters after switch from ACV to BIPAP/APRV mode				
Time between VV-ECMO implantation and switch from ACV to BIPAP/APRV, hours	4 [2–12]	2 [2–4]	10 [6–12]	< 0.0001
Tidal volume, mL/kg PBW	2.5 [1.5–3.5]	2.5 [1.7–3.4]	2.4 [1.2–3.6]	0.7
PEEP, cmH_2_O	12 [8–12]	12 [12–12]	12 [8–12]	0.43
Driving pressure, cmH_2_O	12 [12–12]	12 [12–12]	12 [12–12]	0.75
Respiratory rate, breaths/min	20 [20–24]	22 [20–28]	20 [20–20]	0.02
Plateau pressure, cmH_2_O	24 [24–24]	24 [24–24]	24 [22–24]	0.84

*VV-ECMO*, venovenous extracorporeal membrane oxygenation; *ACV* volume assist control ventilation; *BIPAP/APRV* bi-level positive airway pressure/airway pressure release ventilation; *PEEP* positive end-expiratory pressure; *PBW* predicted body weight

^a^using the last pre-implantation arterial blood gas (ABG) and the first ABG following it

^b^Using the last pre-implantation ABG and all ABG sampled within the 12 h following implantation

### Dual titration protocol

**Fig. 2:**
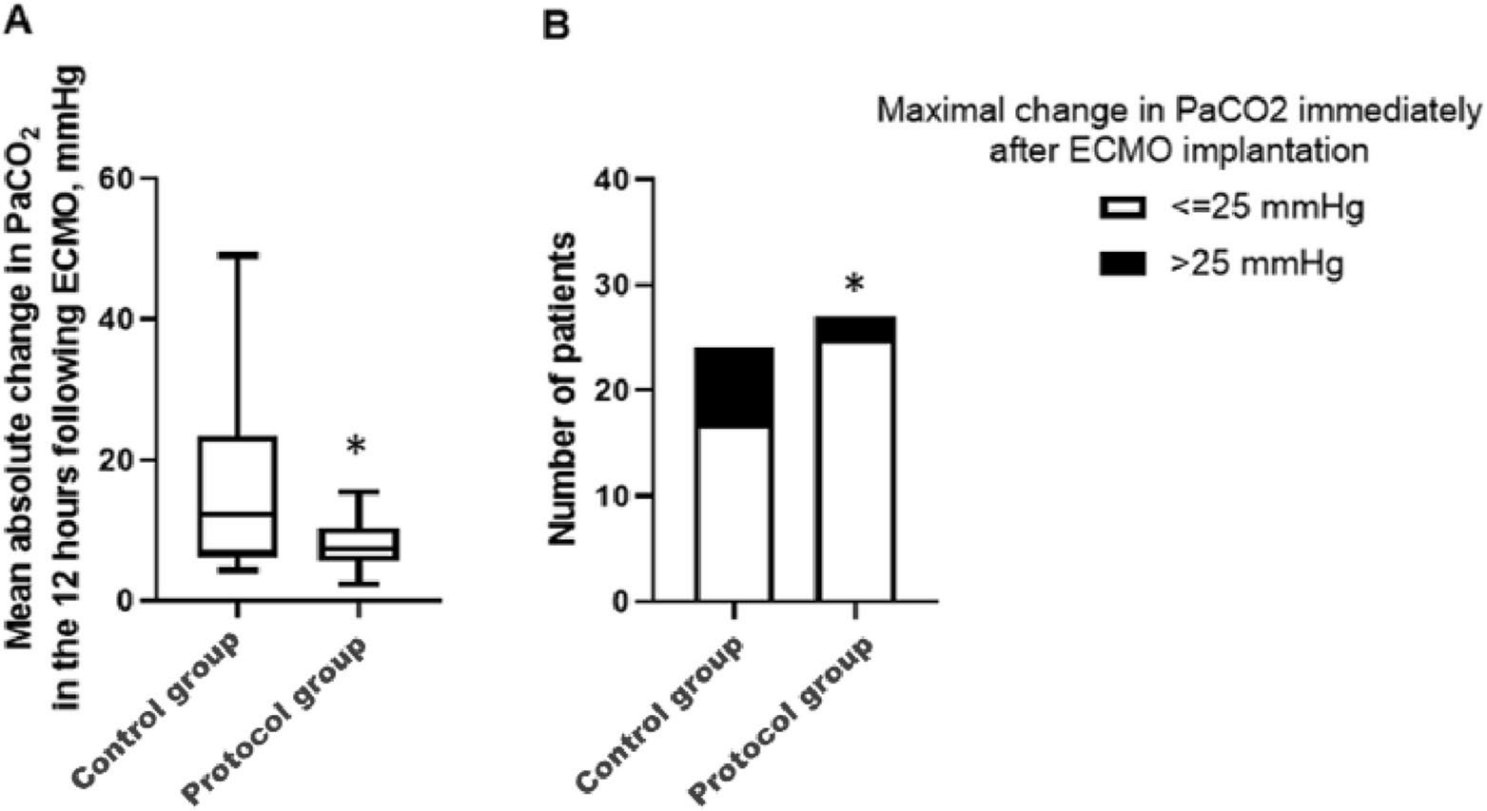
Change in partial pressure of carbon dioxide in arterial blood (PaCO2) in control and protocol groups. **A** Shows mean absolute change in PaCO_2_ calculated using the last pre-implantation arterial blood gas and all arterial blood gas samples drawn within the 12 h following implantation. **B** Shows the number of patients who experienced a change in PaCO_2_ > 25 mmHg, calculated using the last pre-implantation arterial blood gas (ABG) and the first post-implantation ABG by subtracting the pre-implantation PaCO_2_ from the post-implantation PaCO_2_ (in absolute value). *denotes a *p* value < 0.05

The median number of ABG samples was higher in the protocol group than in the control group: 5 [5, 6] vs 4 [1–5], *p* < 0.0001. Ultra-protective ventilation was achieved in a median delay of 10 h (Table 2). Among the 162 needed ABG, 143 (88%) were actually draw during the 12 h following VV-ECMO implantation, and 19 scheduled samplings (12%) were missing (due to omission or lack of time given the work overload). Most of the SGF and ventilator setting adjustments (118/152, 78%) were made in compliance with the protocol (34 changes were not in line with the protocol, of which 9 changes were considered incorrect due to the absence of ABG). Overall, the protocol was deemed feasible as > 75% of scheduled ABG were performed and > 75% of V_M_ and SGF adjustments were in accordance with the protocol. The primary endpoint, i.e. the mean absolute change in PaCO_2_ over the 12 h following VV-ECMO implantation was lower in patients of the protocol group than it was in those of the control group (Fig. 2 and Table 2). The percentage of patients with large PaCO_2_ variations (> 25 mmHg) immediately after VV-ECMO implantation was also significantly reduced in the protocol group (Fig. 2 and Table 2). Of note, the protocol application resulted in less immediate reduction in tidal volume, driving pressure, and plateau pressure after VV-ECMO implantation, and in a slower transition of ventilator settings from ACV to BIPAP/APRV (Table 2).

### Outcomes

The overall rate of bleeding was similar between groups, and patients of the “protocol group” experienced less intracranial bleeding than their counterparts (Table 3), despite similar haemostatic parameters at initiation and during VV-ECMO support (Table 4). The complete characteristics of patients with intracranial bleeding are described in Additional file 1: Table S3. Briefly, patients who bled had comorbidities (e.g., arterial hypertension in all) and no or few anticoagulant overdoses. However, most of them (5/7) experienced significant variations in PaCO_2_.

**Table 3:** Outcome of patients

Variables	All patients (*n* = 51)	Control group (*n* = 24)	Protocol group (*n* = 27)	*p*
VAP	36 (71)	15 (63)	21 (78)	0.23
ICU length of stay (d)	31 [21–48]	26 [16–46]	40 [25–70]	0.05
Duration of mechanical ventilation (d)	28 [14–49]	23 [11–41]	30 [19–56]	0.16
Renal replacement therapy	24 (47)	10 (42)	14 (52)	0.47
ECMO implantation by mobile circulatory assistance unit	21 (41)	5 (21)	16 (59)	0.01
ECMO duration (d)	16 [9–25]	13 [6–19]	21 [12–43]	0.01
Death in hospital	20 (40)	11 (46)	9 (35)	0.52
Cause of death				
Intracranial bleeding	3 (6)	3 (13)	0 (0)	0.1
MOF	16 (31)	7 (29)	9 (33)	0.74
Cardiac arrest	2 (4)	1 (4)	1 (4)	1
Bleeding (ISTH definition)				
Minor bleeding	4 (8)	1 (4)	3 (11)	0.61
Major bleeding	32 (63)	16 (67)	16 (59)	
Site of bleeding				
Number of performed brain CT	0 [0–1]	0 [0–1]	0 [0–1]	0.87
Number of patients who had at least one brain CT during ECMO run	18 (35)	8 (33)	10 (37)	0.78
Intracranial	7 (14)	6 (25)	1 (4)	0.04
ORL	21 (41)	9 (38)	12 (44)	0.62
Gastro-intestinal	7 (14)	3 (13)	4 (15)	1
Lung	3 (5)	1 (4)	2 (7)	1
Cannula	20 (39)	11 (46)	9 (33)	0.36
Tamponade	1 (2)	0 (0)	1 (4)	1

*SAPS 2* Simplified Acute Physiology Score 2; *ICU* intensive care unit; *COPD* chronic obstructive pulmonary disease; *VAP* ventilator acquired pneumonia; *ICU* intensive care unit; *ECMO* extracorporeal membrane oxygenation; *MOF* multiple organ failure; *ISTH* international Society on Thrombosis and Haemostasis

**Table 4:** Haemostatic parameters with ECMO

Variables	All patients (*n* = 51)	Control group (*n* = 24)	Protocol group (*n* = 27)	*p*
ECMO implantation^a^				
Fibrinogen, g/l	7.3 [5.4–8]	7.6 [5.8–8.6]	6.8 [4.8–7.9]	0.29
Platelet count, G/l	264 [197–332]	273 [198–350]	261 [196–311]	0.5
During ECMO support				
Lowest fibrinogen, g/L	3.5 [2.6–4.6]	3.8 [3–5.1]	3.1 [2.3–4.4]	0.05
Lowest platelet count, G/L	111 [63–166]	113 [66–165]	110 [62–171]	0.78
Median anti-Xa on ECMO, IU/L	0.36 [0.28–0.43]	0.39 [0.31–0.48]	0.32 [0.26–0.38]	0.06
Number of day with an anti-Xa > 0.7 IU/L	1 [0–1]	0 [0–1]	1 [0–1]	0.4
Lowest PT, %	57 [47–64]	56 [44–63]	57 [49–70]	0.36

*ECMO* extracorporeal membrane oxygenation; *PT* prothrombin time

^a^Within the 24 h before or after ECMO implantation; anti-Xa in IU/ml

Mortality was similar in the two groups. Patients of the protocol group had a trend towards higher ICU length of stay and a longer duration on VV-ECMO support (Table 3).

## Discussion

This study is the first to propose a pragmatic protocol for dual management of oxygenator SGF and V_M_ in the first hours following VV-ECMO implantation. Our main findings show that the protocol was feasible, achieved the goal of reducing PaCO_2_ variations, and the patients who were put on it had less intracranial bleedings.

Our protocol allowed the switch to ultra-protective ventilation within 10 h of VV-ECMO implantation, which appears acceptable. Furthermore, feasibility and adherence to the protocol was relatively good (88% for ABG sampling and 78% for adequate changes in ventilatory settings and SGF). Due to the retrospective nature of the study, the causes of errors could not be clearly identified. Nevertheless, it likely that these errors were related to a lack of time or an omission of ABG.

Intracranial bleeding in patients managed with VV-ECMO is a strong determinant of mortality [13]. The cerebral vascular tone is highly sensitive to changes in PaCO_2_ [6, 14, 15] and both hypercapnia and hypocapnia have been associated with worse outcomes in patients with neurological injury, in general [16, 17]. Hypercapnia induces vasodilatation with consequent increase in cerebral blood flow with high risk of developing cerebral oedema due to hyperperfusion [18]. A global vasodilatation could cause a steal phenomenon whereby regions with less vasodilatory reserve become hypoperfused, thus suffer focal ischaemia [19]. On the other hand, a rapid decrease in PaCO_2_ potentially reduces cerebral blood flow which compromises brain perfusion and leads to ischaemia as well [20, 21]. Additionally, hypocapnia increases affinity of haemoglobin for oxygen which can compromise oxygen delivery to the brain [22].

Several studies previously demonstrated that following VV-ECMO implantation large initial variations in PaCO_2_ (*i.e.*, ≥ 25 mmHg) were associated with cerebral bleeding [7–9], a complication usually reported in 10 to 20% of patients [4, 7–9, 23]. In our entire cohort, 14% of patients showed intracranial bleeding, which is consistent with previous findings. Most (5/7) of those patients exhibited an initial change in PaCO_2_ of more than 25 mmHg and had a mean change in PaCO_2_ of 24 mmHg within the 12 h following ECMO implantation. This stresses the importance of limiting PaCO_2_ variations after ECMO implantation. Our primary endpoint was thus the mean absolute change in PaCO_2_ within the 12 h following ECMO implantation in order to assess the effectiveness of the protocol over the several hours needed to achieve a steady ventilation and VV-ECMO parameters. We demonstrated that our protocol was effective in reducing PaCO_2_ changes in that setting. Nevertheless, in our study, all patients who bled had already had a past medical history of arterial hypertension, which is in itself a proven risk factor for intracranial bleeding. Owing to the small number of patients, we could not scrutinise the respective roles of hypertension and capnia changes in the occurrence of bleeding in our cohort. Further studies are necessary.

Sustained high values of driving pressure have been associated with a higher mortality in patients on VV-ECMO [24–26]. The implementation of our protocol resulted in slower achievement of the plateau and driving pressure targets. However, whether the additional hours observed in the protocol group to reach ultra-protective ventilation (10 vs 2 h, a bit anecdotal if compared with the 16 days of VV-ECMO support in our cohort) have clinical consequences, is a question that merits further research to weigh the time surplus against the potential reduced risk of intracranial bleeding. Overall, this statement must be taken with caution given the small number of patients in our groups. The main strengths of our study lie in the novelty of the proposed dual titration protocol, and the detailed recording of respiratory and gas exchange variables.

Our study has several limitations. First, it is a monocentric retrospective study with a small sample size. Second, we noted some differences between the groups: patients in the control group were older and often males, while those in the protocol group were often obese, with higher SAPS2 score, and their ECMO was often implanted by our mobile team, a situation where SGF and V_M_ are more difficult to manage during ambulance transport. Third, because we did not routinely perform brain CT under ECMO, we may have underestimated the incidence of intracranial bleeding. However, we did not change our practice of performing brain CT during the study period. Fourth, the inclusion of two groups of patients over two different time periods may lead to a historical bias, especially during the COVID-19 outbreak, a period characterised by rapid change in therapeutic management [27, 28]. For example, some of our patients had benefited from corticosteroids and others had not. However, it is unlikely that COVID-19 therapeutic management affected our primary endpoint. Given the small sample size, we could not match the patients of the two groups. The small number of events also precluded a multi-variable analysis with logistic regression. Eventually, our study included only COVID-19 patients, therefore it is important to evaluate the proposed titration protocol in patients with other ARDS aetiologies.

## Conclusion

Implementation of our protocol for dual titration of minute ventilation and sweep gas flow was feasible and associated with less initial PaCO_2_ variation than usual care. It was also associated with less intracranial bleeding.

## Supplementary Information

**Additional file 1: Table S1.** Mondor dual protocol for adaptation of oxygenator sweep gas flow on the ECMO machine and minute ventilation on the mechanical ventilator after ECMO implantation. **Table S2.** Example of dual titration of mechanical ventilator minute ventilation and oxygenator sweep gas flow in a patient supported by venovenous extracorporeal membrane oxygenation. **Table S3.** Description of patients with cerebral bleeding.

## Data Availability

The dataset used during the current study is available from the corresponding author upon reasonable request.

## Abbreviations

ABG: Arterial blood gas
aPTT: Activated partial thromboplastin time
ARDS: Acute respiratory distress syndrome
ACV: Assist control ventilation mode
BIPAP/APRV: Biphasic positive airway pressure/airway pressure release ventilation
BMI: Body mass index
ICU: Intensive care unit
ISTH: International Society on Thrombosis and Haemostasis
MOF: Multiple organ failure
MV: Mechanical ventilation
PBW: Predicted body weight
PEEP: Positive end-expiratory pressure
SAPS 2: Simplified Acute Physiology Score 2
SGF: Sweep gas flow
VM: Ventilation minute (l/min)
VV-ECMO: Venovenous extra-corporeal membrane oxygenation
