# Supplementary material for: Dual titration of minute ventilation and sweep gas flow to control carbon dioxide variations in patients on venovenous extracorporeal membrane oxygenation
Source: Ann Intensive Care. 2023 May 25;13:45. doi: 10.1186/s13613-023-01138-5 (PMC10208916; doi:10.1186/s13613-023-01138-5)
Supplement: Supplementary file 1 — Additional file 1: Table S1. Mondor dual protocol for adaptation of oxygenator sweep gas flow on the ECMO machine and minute ventilation on the mechanical ventilator after ECMO implantation. Table S2. Example of dual titration of mechanical ventilator minute ventilation and oxygenator sweep gas flow in a patient supported by venovenous extracorporeal membrane oxygenation. Table S3. Description of patients with cerebral bleeding. [file 13613_2023_1138_MOESM1_ESM.zip › table S2.docx]

# Table S2: Example of dual titration of mechanical ventilator minute ventilation and oxygenator sweep gas flow in a patient supported by venovenous extracorporeal membrane oxygenation

|  | Pre-ECMO | H0 | H1 | H2 | H4 | H6 | switch |
| --- | --- | --- | --- | --- | --- | --- | --- |
| **SGF, L/min** | 0 | 1 | 2 | 3 | 4 | 5 | 5 |
| **V_T_, mL** | 300 | 300 | 250 | 187 | 129 | 124 | 111 |
| **RR, pm** | 28 | 28 | 28 | 28 | 28 | 15 | 15 |
| **V_M_, L/min** | 8,500 | 8,500 | 7,000 | 5,236 | 3,612 | 1,860 | 1,665 |
| **PEEP** | 13 | 12 | 12 | 12 | 12 | 12 | 12 |
| **PlatP** | 32 | 31 | 29 | 27 | 24 | 24 | 24 |
| **Ventilation mode** | ACV | ACV | ACV | ACV | ACV | ACV | APRV |
| **PaCO_2_, mmHg** | 54 | 38 | 40 | 40 | 42 | 42 | 41 |
| **ECMO: extracorporeal membrane oxygenation; SGF: sweep gas flow; V_T_: Tidal volume; RR: respiratory rate; V_m_ minute ventilation; AVC: assist control ventilation; APRV: airway pressure release ventilation; H: hour** | | | | | | | |
