# Supplementary material for: Dual titration of minute ventilation and sweep gas flow to control carbon dioxide variations in patients on venovenous extracorporeal membrane oxygenation
Source: Ann Intensive Care. 2023 May 25;13:45. doi: 10.1186/s13613-023-01138-5 (PMC10208916; doi:10.1186/s13613-023-01138-5)
Supplement: Supplementary file 1 — Additional file 1: Table S1. Mondor dual protocol for adaptation of oxygenator sweep gas flow on the ECMO machine and minute ventilation on the mechanical ventilator after ECMO implantation. Table S2. Example of dual titration of mechanical ventilator minute ventilation and oxygenator sweep gas flow in a patient supported by venovenous extracorporeal membrane oxygenation. Table S3. Description of patients with cerebral bleeding. [file 13613_2023_1138_MOESM1_ESM.zip › table S3.docx]

# Table S3: Description of patients with cerebral bleeding

|  | **Control group** | | | | | | **Protocol group** |
| --- | --- | --- | --- | --- | --- | --- | --- |
|  | **Patient 1** | **Patient 2** | **Patient 3** | **Patient 4** | **Patient 5** | **Patient 6** | **Patient 7** |
| Age (years) | 57 | 55 | 57 | 54 | 51 | 58 | 54 |
| Comorbidities | Diabetes mellitus  Hypertension | Diabetes mellitus  Hypertension | Diabetes mellitus  Hypertension | Hypertension | Hypertension | Hypertension | Hypertension |
| ECMO implantation by  mobile circulatory assistance unit | no | yes | yes | yes | no | no | yes |
| ECMO duration (days) | 18 | 26 | 1 | 2 | 17 | 12 | 43 |
| **Hemostasis parameters with ECMO** | | | | | | | |
| Fibrinogen, lowest value (g/L) | 1.4 | 3.2 | 5.7 | 6 | 4.1 | 3 | 4.4 |
| Platelet count, lowest value (G/L) | 89 | 76 | 141 | 273 | 60 | 139 | 147 |
| Anti Xa activity, highest value (IU/mL) | 0.28 | 0.53 | 0.37 | 0.82 | 0.97 | 0.8 | 0.71 |
| Anti Xa activity, mean value (IU/mL) | 0.13 | 0.14 | 0.23 | 0.45 | 0.42 | 0.39 | 0.30 |
| Prothrombin time, lowest value (%) | 53 | 39 | 53 | 73 | 38 | 58 | 46 |
| Days with anti Xa > 0.7 | 0 | 0 | 0 | 1 | 1 | 0 | 1 |
| Anti Xa activity before bleeding, mean value (IU/mL) | 0.15 | 0.33 | 0.23 | 0.58 | 0.42 | 0.39 | 0.3 |
| Anti Xa activity before bleeding, highest value (IU/mL) | 0.24 | 0.53 | 0.37 | 0.82 | 0.97 | 0.69 | 0.71 |
| Days with anti Xa > 0.7 before bleeding | 0 | 0 | 0 | 1 | 2 | 0 | 1 |
| **Bleeding characteristics** | | | | | | | |
| Number of brain CT-scans | 3 | 2 | 1 | 1 | 2 | 1 | 1 |
| Days on ECMO at first CT-scan | 4 | 4 | 2 | 2 | 2 | 11 | 41 |
| Days on ECMO at CT-scan with bleeding diagnosis | 4 | 4 | 2 | 2 | 17 | 11 | 41 |
| Symptom leading to CT-scan and bleeding diagnosis | myoclonia | anisocoria | loss of brain stem reflexes | mydriasis | none (systematic full body scan) | anisocoria | none (systematic full body scan) |
| Site of intracranial bleeding | Diffuse subarachnoid haemorrhage | Right occipital haematoma 22 mm with peri-lesional oedema, subarachnoid haemorrhage. | Left temporo-occipital haematoma, right cerebellar haematoma, subarachnoid haemorrhage. | Right frontal subdural haematoma, right parieto-occipital intracerebral haematoma, bilateral fronto-parietal, subarachnoid haemorrhage. | Right temporal and occipital subarachnoid haemorrhage. | Right fronto-parietal haematoma, 60 mm. | Left occipital intraparenchymal haematoma 37 x 18 mm with peri-lesional oedema. |
| CT illustration of bleeding | 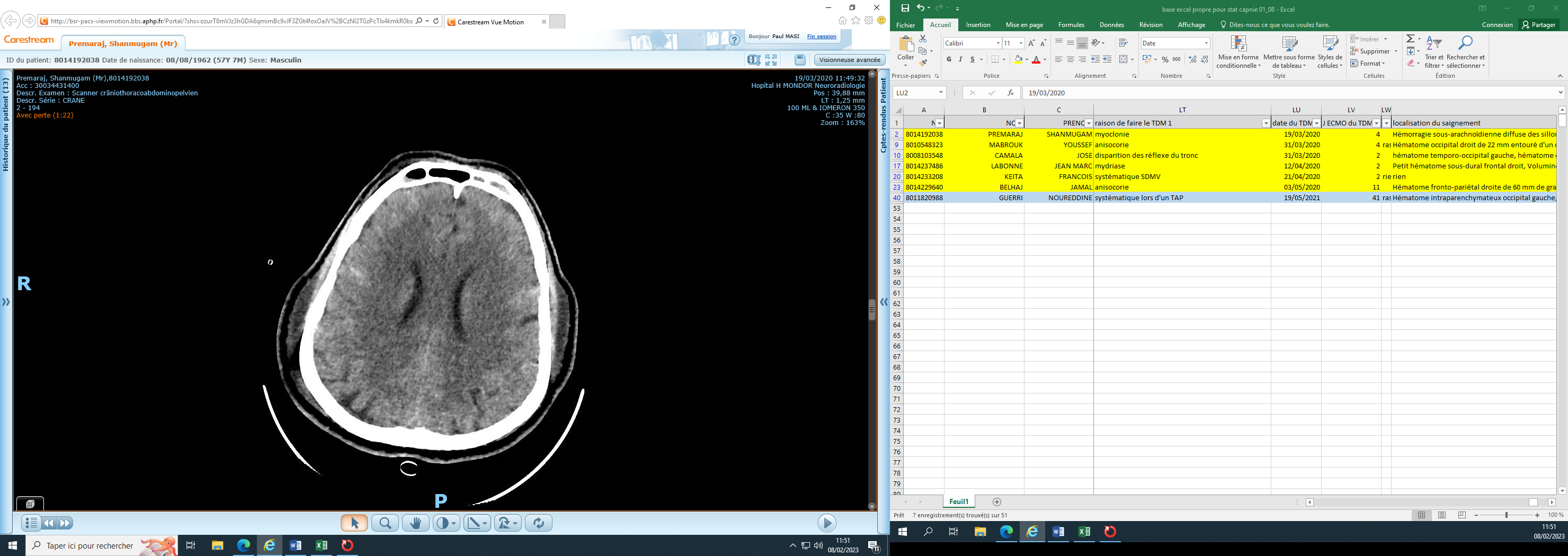 | 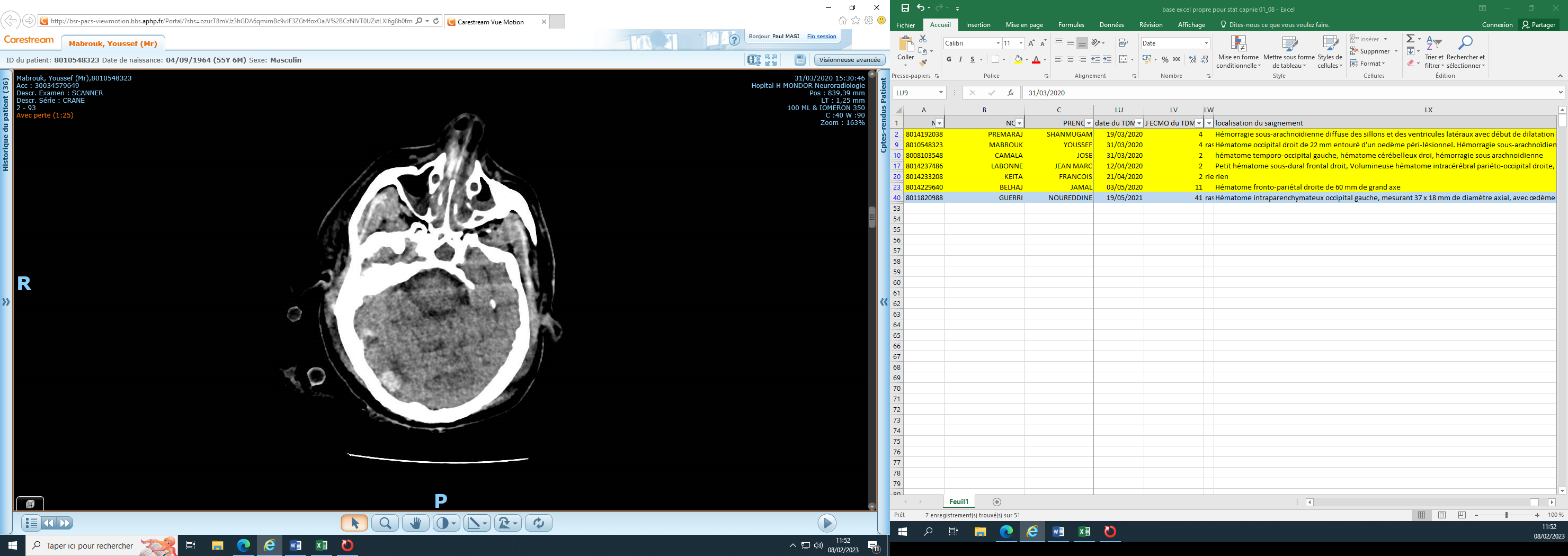 | 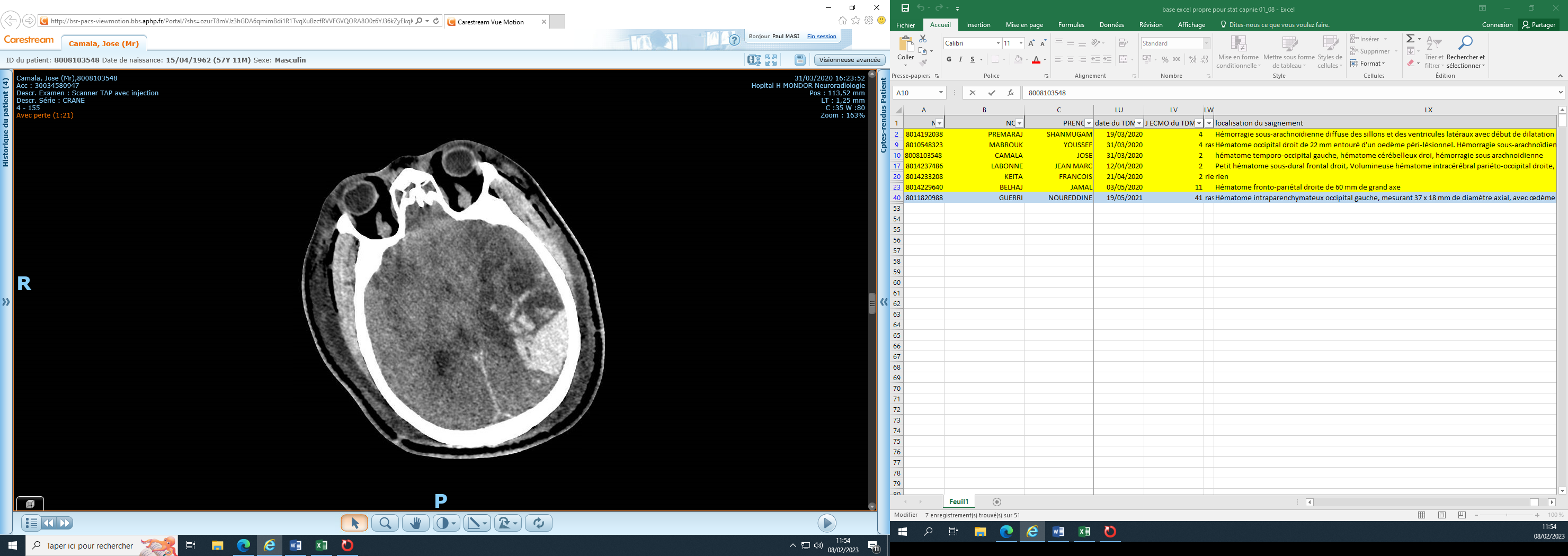 | 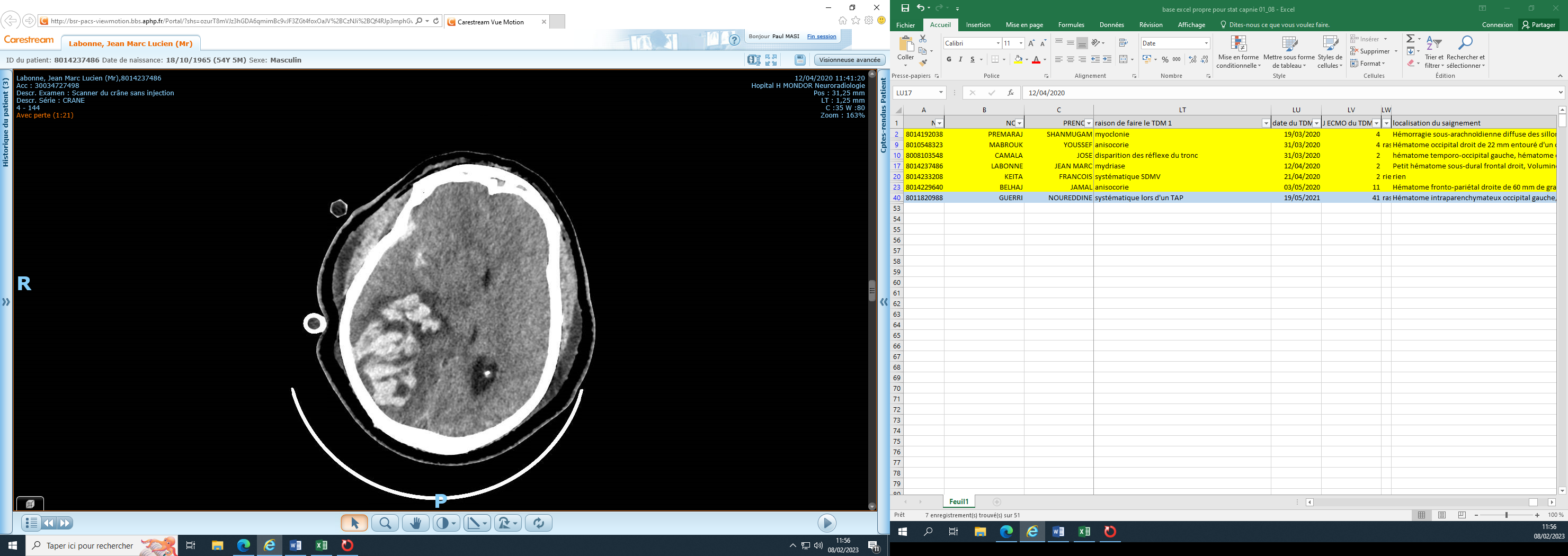 | 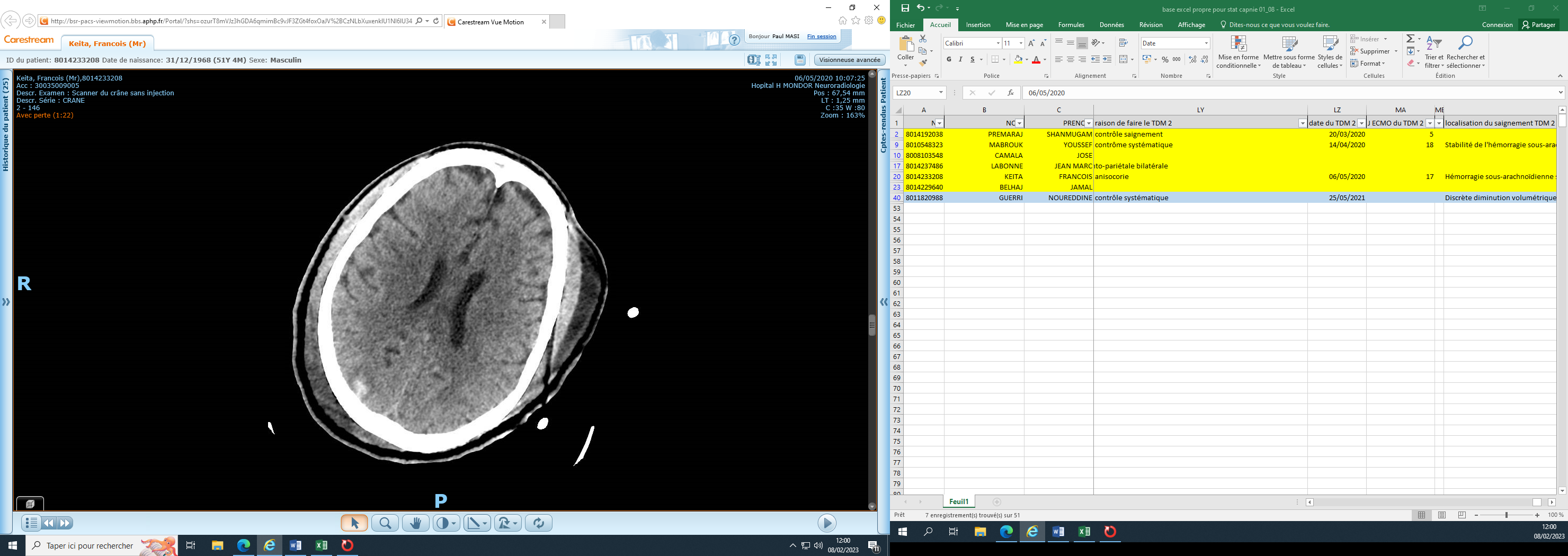 | 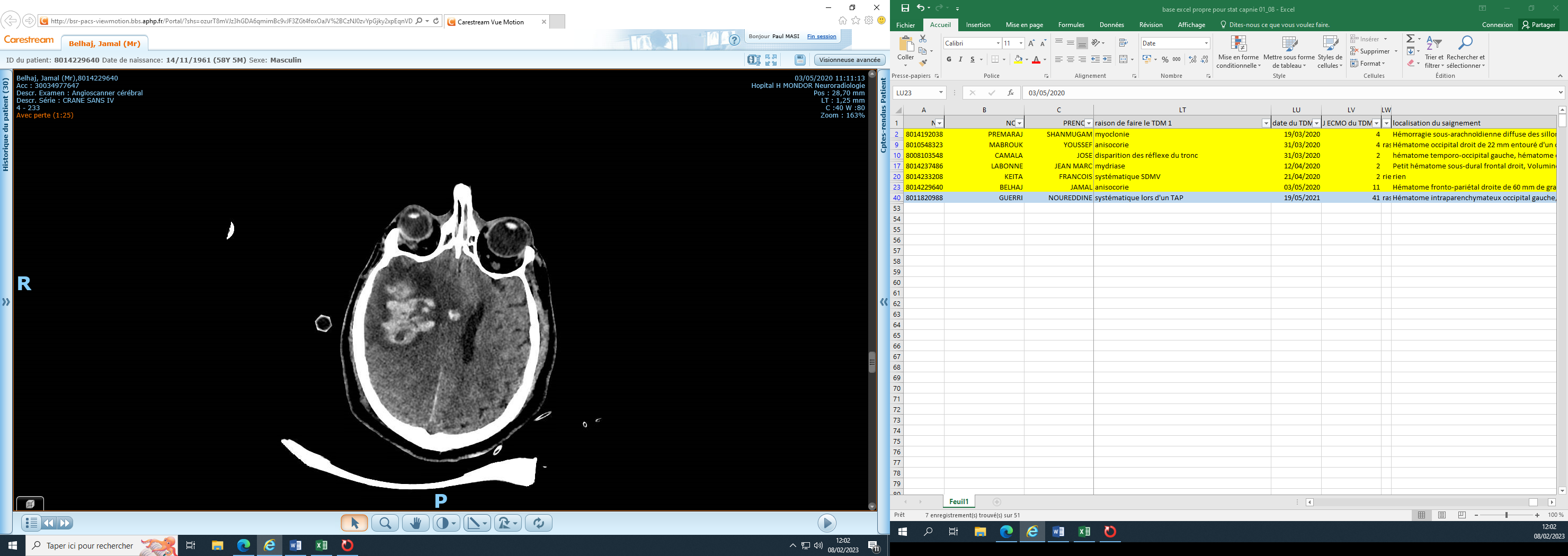 | 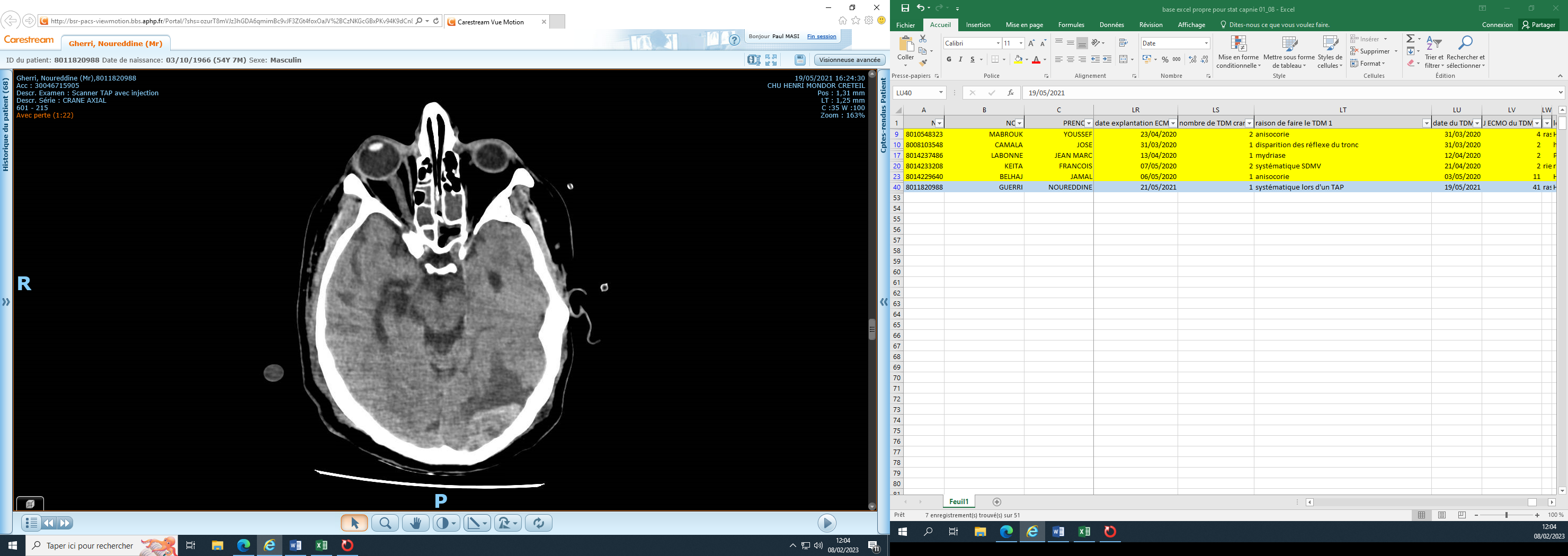 |
| Death | yes | no | yes | yes | yes | yes | no |
| Cause of death | Septic shock and multiple organ failure | - | Brain death | Brain death | Limitation of active treatments and multiple organ failure | Brain death | - |
| **Capnia variation** | | | | | | | |
| Absolute change in PaCO_2_ just after ECMO implantation > 25 mmHg* | no | yes | yes | yes | no | yes | yes |
| Mean absolute change in PaCO_2_ within the 12 hours following ECMO implantation# (mmHg) | 24 | 26 | 19 | 30 | 6 | 49 | 11 |
| **VV-ECMO, venovenous extracorporeal membrane oxygenation; ACV, volume assist control ventilation; BIPAP/APRV, bi-level positive airway pressure/airway pressure release ventilation; *using the last pre-implantation arterial blood gas (ABG) and the first post-implantation ABG; # using the last pre-implantation ABG and all ABG sampled within the 12 hours following implantation; PEEP: positive end expiratory pressure; ECMO: extracorporeal membrane oxygenation; Anti Xa in IU/ml; PT prothrombin time.** | | | | | | | |
